# Success Rates and Complications Associated with Single Immediate Implants: A Systematic Review

**DOI:** 10.3390/dj10020031

**Published:** 2022-02-21

**Authors:** Charn Thanissorn, Jason Guo, Dianna Jing Ying Chan, Bryar Koyi, Omar Kujan, Nabil Khzam, Leticia Algarves Miranda

**Affiliations:** 1 UWA Dental School, The University of Western Australia, 17 Monash Ave, Nedlands, WA 6009, Australia; 21480116@student.uwa.edu.au (C.T.); 21497777@student.uwa.edu.au (J.G.); 21490549@student.uwa.edu.au (D.J.Y.C.); omar.kujan@uwa.edu.au (O.K.); nabil.khzam@research.uwa.edu.au (N.K.); 2 Independent Researcher, London W11 3LF, UK; bryarkoyi@hotmail.com; 3 Independent Researcher, Como, WA 6152, Australia

**Keywords:** dental implant, immediate placement, success rate, complications, systematic review

## Abstract

This study examined the success rates of single immediate implants and their associated biological, hardware and aesthetic complications. Using a developed search strategy, randomized controlled trials (RCTs) on single-unit immediate implants with at least six human participants, a minimum follow-up time of 12 months and published between January 1999 and January 2021 were identified. Data was extracted independently using pre-designed data extraction forms. Information on success rates and associated biological, hardware and aesthetic complications were obtained and assessed. Out of 191 potentially eligible studies, 26 RCTs assessing 1270 patients with a total of 1326 single implants were included and further evaluated. In this review, success rate was reported to be 96.7–100% over a total of 9 studies. However, there was a lack of consensus on a universal success criterion between authors emphasizing the need for agreement. The average follow up was 29 months and most reported complications were aesthetic (63 cases, 4.7%), whilst there were relatively fewer biological, (20 cases, 1.5%), and hardware complications (24 cases, 1.8%). Success rate is an uncommon clinical outcome with 9 out of 26 of the selected RCTs reporting it. In these studies, single immediate implants showed a high success rate with low numbers of biological and hardware complications, and high patient satisfaction with aesthetics were reported in the short-term follow-up of one year.

## 1. Introduction

Modern implant dentistry aims at improving patient masticatory function, aesthetics, and overall quality of life. Dental implants achieve these aims via the concept of osseointegration. In 1969, Per-Ingvar Branemark described osseointegration as “a direct structural and functional connection between ordered living bone and the surface of the load-covering implant” [1]. Nowadays, more advanced techniques and methods to compensate for tooth loss in patients using immediate implantation are implemented. Immediate implant placement involves placing dental implants into fresh extraction sockets (IIP). Traditionally dental implants were placed into healed sites with a two stage approach; conventional implant placement (CIP), however this came at the cost of increased length in treatment time and multiple surgical procedures to patients [2,3]. 

IIP was first reported by Schulte et al. and since then advances have been made in implant materials, soft and hard tissues augmentation techniques of tooth extraction sites [4]. Studies show that IIP can provide similar success rates to delayed implant placement protocols with some showing the IIP protocol being preferred by patients [5,6,7,8]. IIP has also been shown to achieve greater efficiency in treatment time, and patient comfort compared with CIP [2,3]. Several papers also report that there is no significant difference in peri-implant marginal bone levels between IIP and CIP [5,8].

The difficulty in comparing success between different implant placement protocols is in the definition of ‘success’ as there is no consensus on a criterion for success. Authors would develop their own success criteria leading to confusion or lack of clarity when comparing treatment outcomes in the literature [9,10]. Another issue is that the majority of papers report survival as a primary outcome with Needleman et al. reporting 60% of 216 included studies used survival as the primary outcome compared with success at 15.7%. Another systematic review compared IIP with CIP, concluding that a few studies report on success rates [11]. Tonetti et al. reported that ‘outcome measurements related to implant-supported rehabilitation cannot be limited to implant survival or success rates, however, when appropriate should also include the functional performance and aesthetic aspects of the entire rehabilitation as well as the health status of the peri-implant tissues” [12]. In the current literature of implant dentistry there are limitations to reporting success as an outcome including the lack of consensus amongst authors on a definition, a lack of consensus on outcomes that measure success, variations in the timing of outcome assessments, and a lack of details in reporting.

Clinically an implant should not only survive but be successful with no biological, hardware or aesthetic complications. One systematic review reported on the survival rate, success rate and complications of IIP, both in single and in multiple implant cases [13]. The study concluded a high survival rate for IIP, however did not report the success rate. They also reported complications with midfacial gingival recession, resulting in suboptimal aesthetic results for IIP with delayed restoration, yet found only one paper that reported on aesthetics using Pink Esthetic Score (PES), White Esthetic Score (WES) or Implant Crown Aesthetic Index (ICAI). A Cochrane systematic review evaluated the success and complications associated with IIP, immediate-delayed, and CIP using RCTs [3]. The authors concluded there was insufficient evidence to determine the possible advantages and disadvantages amongst the different timing protocols for implant placement, and that current conclusions are only based on studies that are underpowered and are often at a high risk of bias.

This systematic review aims to provide an updated insight into the success rates and complications associated with IIP. This will be the first systematic review that looks at the success of single IIP whilst using randomized control studies.

The objectives of this systematic review were to (1) determine a success rate for single IIP when reported, and (2) to provide an updated exploration of the possible biological, hardware, and aesthetic complications associated with IIP.

## 2. Materials and Methods

The review protocol was registered with the International Prospective Register of Systematic reviews (PROSPERO CRD215810). The reporting was carried out following the guidelines of the Preferred Reporting Items for Systematic Reviews and Meta-Analysis (PRISMA) [14]. 

### 2.1. Search Strategy

A detailed electronic search strategy was developed to search PubMed, Embase and Scopus databases to identify all articles published in relation to the stated aims of this review. In addition, a manual search of the Clinical Oral Implants Research and European Journal of Oral Implantology was attempted for the past 10 years to identify any relevant studies. The search was complemented by hand-searching the references of the selected articles for additional publications. The search strategy consisted of a combination of free text terms searched from January 1999 to 1 January 2021. The following search strategy was used: (maxilla OR mandible OR anterior OR posterior) AND (implants OR extraction OR immediate OR placement OR single OR loading OR survival OR success OR complications OR failure OR biological complication OR peri implantitis OR aesthetic complications OR bone loss OR gingival recession) AND (dental implants OR single tooth OR immediate implants OR immediate implant placement OR extraction socket OR immediate implant installation OR single immediate implant placement) NOT (case report) NOT (case series) NOT (full arch) NOT (fixed prosthesis).

### 2.2. Focused Question

Our focused question was defined as “what is the success rate of single immediate implants and what are the associated complications?” The research question was elaborated according to the PICO format [15], where:

Participants: Subjects requiring a single implant in the maxillary and mandibular arches;

Intervention: Implant is placed immediately in an extraction socket in either anterior or posterior regions (placement protocol Type I) [16];

Comparison: Longitudinal follow-up for the replacement of a single tooth in the maxillary and mandibular region;

Outcomes: Implant success rate, complications. 

The elected success outcomes of this study were based around the statements produced by the Third ITI Consensus Conference [17]. The timing of implant placement post tooth extraction was defined as follows; type-I being immediate post extraction placement, type-II is 4-to-8-week placement, type-III is 12-to-16-week placement and type IV being more than 16 weeks. This study evaluated success and complications associated with single implant type-I placements.

The absence of any biological, hardware, or aesthetic complications, and the presence of the dental implant or reconstruction were defined as implant success.

Biological complications refer to pathological changes in the surrounding peri-implant tissues. The signs of complications can include the following; inflammation, bleeding on probing, suppuration, increased probing depths compared to baseline levels, and bone loss beyond initial remodeling [17]. The two main biological complications affecting implants are peri-implant mucositis and peri-implantitis. Peri-implant mucositis is defined as clinical signs of inflammation in the peri-implant mucosa usually indicated by bleeding on gentle probing [18,19]. Peri-implantitis refers to clinical signs of inflammation as in the case of peri-implant mucositis in addition to progressive loss of supporting bone [18]. Other conditions may develop after implant placement that can be classified as biological complications include medication-related osteonecrosis of the jaw, oral mucosal disorders, allergies, and implant-related tumors.

Hardware complications can be split into two categories—technical and mechanical. Technical complications are those that affect the laboratory-fabricated component of the prosthesis, like veneering material fracture, crown fracture, framework fracture and loss of retention of the prosthesis. Mechanical complications are those that affect the pre-fabricated component of the prosthesis, those affecting the implant itself or the associated abutment and occlusal screws. 

Aesthetic complications are those that affect the aesthetics of the peri-implant soft tissues, of the implant crown itself, and of the patient satisfaction. Soft tissue aesthetics can be evaluated through many different means, including the following methods that were used in our final selected studies:

- Pink Esthetic Score (PES): Where the score was based on seven parameters: mesial papilla, distal papilla, soft tissue level, soft tissue contour, alveolar process deficiency, soft tissue color, and texture. Each parameter was assessed with a 2–1–0 score, with 2 being the best and 0 being the worst result. A maximum score of 14 can be achieved [20].
- Papilla Index Score (PIS): This includes index-0 means no papilla present, index-1 means less than one half the papilla height is present and a convex nature of the adjacent tissue nature is noted, index-2 means greater than half the height of the papilla is present although not to the full extent of the contact point, papilla is not in complete harmony, index-3 means the papilla fills the entire proximal space and is in good harmony, and finally, index-4 means the papilla is hyperplastic [21].
- Midfacial gingival level: Which can be measured as the most apical portion of the midfacial peri-implant mucosa. Change over time/recession is determined by comparing to a baseline measurement (pre-operatively or to an untreated adjacent tooth).

Implant crown aesthetics can also be assessed via different indices including:

- White Esthetic Score (WES): Where the score includes five variables: Tooth form, tooth volume, tooth color including the assessment of hue and value, tooth texture, and translucency. Each parameter is assessed with a 0–1–2 score with 2 being the best and 0 being the worst score. A maximum score of 10 can be reached [22].
- Implant Crown Aesthetic Index (ICAI): Is an index based off nine variables where penalty points are assigned if not matching to the desired situation: one penalty point for minor (slight) deviations and five penalty points for major (gross) deviations. Zero penalty points means excellent; one or two points means satisfactory; three or four points means moderate; five or more points means poor aesthetics. The nine categories are: mesiodistal dimension of the crown, position of the incisal edge of the crown, labial convexity of the crown, color and translucency of the crown, surface of the crown, position of the labial margin of the peri-implant mucosa, position of mucosa in the proximal embrasures, contour of the labial surface of the mucosa, color and surface of the labial mucosa [23].

Patient satisfaction of the aesthetic outcome can also be evaluated from patient interviews or questionnaires.

### 2.3. Eligibility Criteria

Studies included had to meet the following criteria:

- Publications between January 1999 and January 2021.
- Randomized controlled trials of immediate single implants.
- Sample size of no less than six human subjects.
- Minimum follow up period of one year.
- Studies published in English.

### 2.4. Study Selection

After undertaking the electronic search of included databases by two authors (N.K., B.K.), titles and abstracts of all resultant citations were scanned independently by four reviewers (D.C., C.T., J.G., N.K.). Thereafter, selected abstracts were reviewed to determine selection of full text articles after applying the above eligibility criteria. The full texts of all studies of possible relevance were then obtained for independent review and assessment by the four reviewers (D.C., C.T., J.G., N.K.). All studies meeting the inclusion criteria then underwent data extraction. Studies rejected at this, or subsequent stages were removed and reasons for exclusion recorded. 

### 2.5. Data Extraction

Data was extracted independently using designed data extraction forms (D.C., C.T., J.G., N.K.). Any disagreement was discussed, and a fifth review author consulted where necessary (L.M.). Data was extracted on success and complications reported from the included studies as outlined above.

### 2.6. Risk of Bias Assessment

The quality of the included studies was assessed by six independent reviewers following the Cochrane Risk of Bias tool for randomized controlled trials [24]. This tool encompasses seven criteria: random sequence generation; allocation concealment; blinding of participants and personnel; blinding of outcome assessment; incomplete outcome data; selective reporting and other bias. All studies were judged against these criteria as having low, unclear or high risk of bias. The overall risk of bias was low if all criteria were at low risk of bias, although it was unclear if there was at least one criterion with unclear risk of bias, and high if there was at least one criterion with a high risk of bias. 

## 3. Results

**Figure 1:**
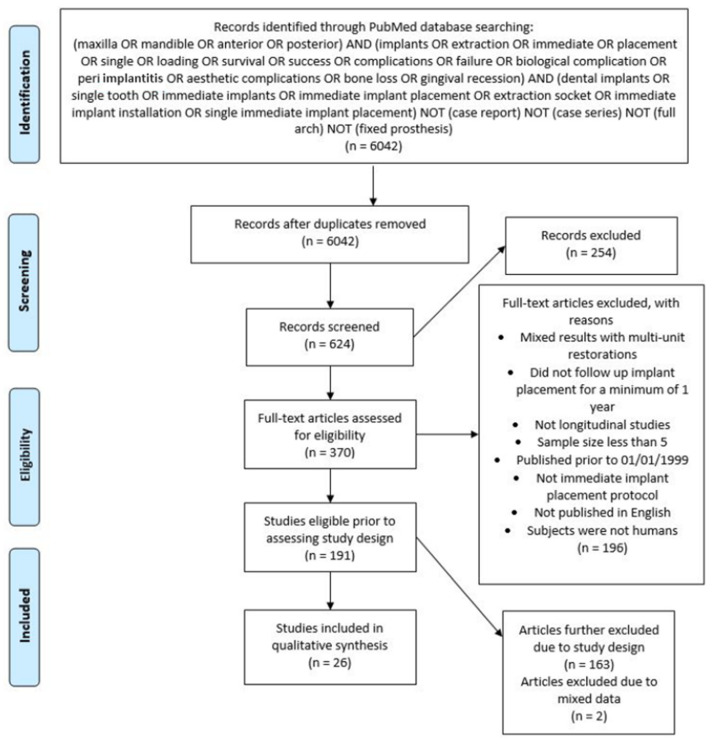
PRISMA flow chart of the screened and included studies.

Of the 6042 titles and abstracts screened, 370 were selected for further analysis (Figure 1). Manual searching of the references lists added 17 studies. Twenty-six randomized control trial studies remained after full-text analysis by the reviewers. A total of two publications reported data on the same subject samples, thus one study with ten years of observation [25] was grouped with its two year counterpart [26].

### 3.1. Risk of Bias within and across Studies

Table 1 summarizes the risk of bias assessment for all included studies. Five studies were deemed an overall low risk of bias, ten were classified an unclear risk of bias and eleven studies were high risk of bias.

### 3.2. Study Characteristics

A total of 1270 patients with 1326 total implants were analyzed. Table 2 outlines the characteristics of the 26 included RCT studies, from which the data was extracted. The follow-up periods of the included studies ranged from 12 to 60 months long with an average of 29 months. There was only one study reporting a five year follow up [25]. Eleven studies were conducted in a university environment [27,28,29,30,31,32,33,34,35,36,37], twelve were private practice or multicenter [25,38,39,40,41,42,43,44,45,46,47,48], and three did not provide enough information for the study setting [49,50,51]. 

### 3.3. Success Rates

Of the 26 included studies, most studies did not define implant success criteria or report a success rate of IIP. A total of nine studies reported a defined success criteria [25,29,31,32,33,34,36,37,41,50] with success rates ranging from 96.7–100%. From these, three studies used a protocol based on radiographic analysis as proposed by Albrektsson et al. [32,37,41,52]. Another three studies utilized their own methods of defining success [25,34,50]. The Smith and Zarb criteria was shared by two studies (no radiolucency or significant marginal bone loss around the implant, no mobility, and no suppuration, discomfort, pain, or neurosensory alteration) [53]. One study determined success following the International Congress of Oral Implantologists, Pisa Consensus Conference, which defined success as no pain or tenderness upon function, no mobility, less than 2 mm radiographic bone loss from initial surgery and no exudates history [9,29]. There were three studies that outlined success criteria yet did not appear to report an enumerative success rate [29,34,50]. The studies, which defined success and characteristics of the success criteria, are summarized in Table 3.

### 3.4. Biological Complications

There were twenty cases of soft tissue complications across six studies [31,33,42,43,46,51]. These complications were peri-implant mucositis, sensory disturbances, edema, and acute infection. Across all studies there was no report of peri-implantitis occurrence. Four studies had biological complications in the posterior region [31,33,42,51], one study in the anterior region [43], and one study from second premolar to second premolar in the maxilla [46]. A summary of the studies and associated biological complications can be found in Table 3.

Two papers reported a case of peri-implant mucositis [42,46]. The first paper compared the IIP protocol with augmentation using a resorbable barrier alone or a bone substitute plus a resorbable barrier. The implants were loaded with provisional or definitive single crowns 3–4 months after implant placement. Peri-implant mucositis occurred around an exposed cover during healing of thirty-six implants in the bone substitute plus resorbable barrier group, and this was treated with chlorhexidine gel. The other study examined the use of immediate restoration using either a platform-switched provisional titanium abutment or a definitive platform-switched titanium abutment [46]. After one year of follow up, one patient in the definitive abutment group (1/12, 8.3%) experienced peri-implant mucositis. The complication was successfully treated.

In addition to the above two cases, four other studies reported biological complications using an immediate placement and delayed loading approach. There was one report of a complication in an anterior implant where a patient received a single square-threaded tapered implant placed using IIP and was immediately restored [43]. One patient experienced moderate sensory disturbances in the labial mucosa in addition to edema three weeks after the surgery. Adjacent teeth also had partial loss of thermal sensitivity. The patient was subsequently treated with 40 mg of bromeline three times daily for one week, after which sensation was restored along with resorption of the edema. One study compared immediate loading to delayed loading after three months and reported an implant failure in the delayed loading group due to acute infection occurring within two weeks of IIP [33]. Urban et al. observed that in 92 patients receiving an immediate implant, 41 developed a soft tissue dehiscence before surgical re-entry, leaving part of the cover screw exposed to the oral environment. In total, thirteen of these patients then developed a postoperative infection at the implant site. A statistically significant proportion of those that developed a soft-tissue dehiscence and subsequent infection were smokers [51].

Pieri and co-workers reported one biological complication using IIP and immediate restoration [31]. The implant failed due to an abscess associated with a fistula and bacterial contamination of the extraction socket. Grandi and colleagues was the only other publication that reported biological complication (peri-implant mucositis) using an immediate restoration technique [46].

### 3.5. Hardware Complications

A total of twenty-four hardware complications across the seven studies were reported [27,31,36,42,44,45,46]. The reported complications included loss of definitive crown, provisional crown fracture, loosening, excess cement, unscrewed occlusal screws, cover screw loosening, provisional and definitive abutment loosening. No hardware complications were reported in the anterior region, whereas five complications were reported in the posterior region. A summary of the studies and associated hardware complications can be found in Table 3. The complications have been further split below into technical and mechanical outcomes. 

### 3.6. Technical Complications

There was a total of fifteen cases of complications that affected laboratory-fabricated component of the prosthesis in six studies [31,36,42,44,45,46]. De Angelis et al. reported loss of retention of one definitive crown, with this study utilizing immediate placed but delayed loaded implants [42]. Esposito et al. reported four cases of provisional crown fracture, and two cases of provisional crown loosening in a group that received immediate implants and immediate loading [44]. Felice et al. reported one case of provisional crown loosening in a patient that had an immediate implant placed and immediately restored, which occurred three months after loading [45]. Four patients in the study conducted by Grandi et al., had excess cement that had to be removed prior to final crown cementation, with all cases being immediately placed and immediately restored [46]. Pieri and co-workers looked at immediately placed and restored implants and reported only one provisional crown fracture after only three weeks post-insertion [31]. Another study that looked at immediately placed and restored implants, by Yoshino et al. reported one case of provisional restoration debonding and one provisional restoration fracture [36]. Only Pieri and co-workers specified whether the complication occurred in the anterior or posterior region—having occurred in the maxillary premolars. 

### 3.7. Mechanical Complications

There was a total of nine cases of complications that affected manufacturer-fabricated components over four studies [27,31,42,46]. Crespi and co-workers compared immediately and delayed loaded immediate implants and reported four unscrewed occlusal screws in the provisional plastic abutments provided in the immediately loaded group [27]. De Angelis and others reported three total mechanical complications in their immediately placed, delayed loaded implants—two incidences of cover screw loosening at 4–6 weeks postoperatively and one incidence of provisional abutment loosening [42]. Grandi et al. reported one patient having an abutment screw loosening—with this case being immediately placed and restored [46]. In another study, Pieri et al. also reported a case of abutment screw loosening two months after delivery of the definitive crown in their immediately placed and restored cases [31]. Of these studies, only two studies specified whether the complication occurred in the anterior or posterior region. De Angelis et al. had all three mechanical complications occurring in posterior teeth, both maxillary and mandibular [42], whilst the single complication found by Pieri et al. occurred in a maxillary posterior tooth [31]. 

### 3.8. Aesthetic Complications

The majority of complications that were reported in association with single IIP were aesthetic complications. Ninety-nine cases of complications associated with PES, WES, ICAI, poor midfacial gingival aesthetics and poor papillary fill were reported across seven studies. Across the studies that reported on patient satisfaction with aesthetics, the outcomes were all high. Of these papers, all but two reported aesthetic complications or patient outcomes from first premolar to first premolar or second premolar to second premolar in the maxilla. The two papers that reported on all regions did not specify a location in the mouth when reporting results [33,42]. A summary of the studies and associated aesthetic complications can be found in Table 3. The complications have been split below into the different aesthetic outcomes.

### 3.9. Clinical Aesthetic Outcome (PES/WES/ICAI)

Eight studies used PES/WES/ICAI as a means to determine aesthetic outcome [34,35,37,42,44,45,48,50]. Mean PES scores ranged from 7.15–13.0 in all eight studies. In the three studies that reported on WES, the mean total WES score reported in these ranged from 6.9–8.1 [34,37,50]. Only one study used ICAI and the scores reported were 4.2 and 5.2 for immediate restoration and delayed restoration of immediate implants, respectively [34]. There were no statistical differences in results when comparing protocols of implant placements amongst these studies. Aesthetic complications were reported by three studies totaling 18 implant cases relating to peri-implant mucosa complications and 11 cases relating to the implant crown [34,37,50]. The rate of clinically unacceptable aesthetic outcome ranged from 0–21.3% in these three studies. Zuiderveld and others reported unacceptable PES and WES scores in 21.3% of the cases across both groups that followed immediate placement and immediate restoration protocol, one without connective tissue graft (CTG) and the other with, and they did not find any statistical differences between total scores and separate items between the groups [37]. Migliorati and co-workers reported on a similar protocol testing tissue augmentation and found that those without tissue augmentation reported PES complications rates of 17.3%, whereas those with augmentation had no PES complications [50]. The Zuiderveld study also tested immediate placement and immediate restoration against immediate placement and delayed restoration, and found a total complication rate of 6% although the study did not report which group the unacceptable scores belonged to and, furthermore, also reported that there was no statistical difference between overall PES/WES/ICAI scores between the groups [34]. Assessment of PES/WES/ICAI was made off photographic records at the final time of recall for all three studies.

### 3.10. Midfacial Gingival Change 

Midfacial gingival levels and recession, when not being assessed by PES, was reported as a measured value comparing the most apical portion of the midfacial gingival level to either a pre-operative baseline or an adjacent tooth. Most of the studies reported a mean value for change in midfacial gingival level (REC) and thus instances of complications were difficult to report from these results [25,28,29,30,31,34,36,37,40,43]. In regard to a change of midfacial gingival levels over time, the mean values ranged from +0.23 mm to −1.16 mm, where a positive value is a gain in gingival height and a negative value signifying a loss in gingival height for these studies. Midfacial gingival levels were only reported as a complication that needed intervention in one study [33]. The study placed implants in both anterior and posterior regions, however, did not report which location the complications occurred in. The study also compared delayed loading with immediate loading of immediate implants, reporting 24.1% (7 implants) with decreased attached gingiva in the delayed group, and 11.5% (3 implants) in the immediate loading group [33] (see Table 3).

### 3.11. Papillary Height

Complications arising from abnormalities in papillary height were represented in few studies using the PIS score. The percentage of patients that had a papillary fill less than 50% (score zero or one) ranged from 13.9–24.4% of total sites across four studies that reported PIS [30,36,37,39]. Of those that scored a PIS of zero or one, 26 were reported to be mesial papilla and 32 were distal papilla. A total of three of the studies placed implants using the IIP and immediate restoration protocol. Results varied from 16.7% [30], 22.5% [36], and 15.5% [37] of patients with papillary height issues. Cecchinato and Palatella studies also reported on immediate placement and delayed restoration and found that 24.4% and 11.7% of these patients had a PIS score of zero or one, respectively [30,39]. All four studies assessed PIS clinically from direct intraoral measurement as opposed to photographic assessment. 

### 3.12. Patient Satisfaction

Seven studies reported on patient satisfaction for the aesthetic outcome [28,34,35,37,42,44,45]. Three of these studies used a similar criterion for evaluating, which was based on the patients answering if they were ‘completely satisfied’, ‘partly satisfied’, ‘not sure’, or ‘absolutely not satisfied’. The proportion of patients reporting complete satisfaction ranged from 94.1–100% in these studies [42,44,45]. The remaining studies reported patient satisfaction based on a 10 cm/100-point VAS scale. Mean satisfaction scores in these immediately placed implants ranged from 82–96% [28,34,35,37]. All six papers reported high satisfaction for aesthetics of IIP, regardless of the protocol used. De Rouck and co-workers compared immediate restoration and delayed restoration in IIP and found no statistical difference between patient satisfaction [28]. Two of the studies compared immediate placement with conventional delayed placement; patient satisfaction of aesthetic outcome was statistically equivalent with both protocols reporting complete satisfaction at 98% and 100% [44,45]. Only one study reported a difference in satisfaction between groups, and that was amongst those that were immediately restored versus delayed, with the delayed group providing higher satisfaction [34].

## 4. Discussion 

A total of eighteen of the twenty-six studies provided data related to a success rate or an associated complication. Follow-up ranged from 1 to 4 years. Success rates were found to have yielded a range from 96.7% to 100% over a total of nine out of the twenty-six studies, utilizing a range of different criteria protocols. A total of 107 complications were reported over 14 studies, with a greater proportion being related to the implant’s aesthetic outcomes. 

### 4.1. Success

Several different criteria for determining implant success have been proposed in the existing literature. Some studies used existing criteria, whilst some even used their own criteria. To this end, success rates in the included studies may have been mistakenly reported than if standardized success criteria were used as not all types of complications may have been accounted for. Thus, it was impossible to provide conclusive evidence on success rates given the lack of a denominating success criteria.

### 4.2. Biological Complications

This review revealed that few studies diagnosed cases of peri-implant mucositis for both IIP and CIP protocols whilst there were no reported cases of peri-implantitis. There is no review at the current time of writing this paper that examines the prevalence of peri-implant disease in IIP. One past study treated 294 patients with CIP therapy and recalled them at one- and five-year intervals. In total, 218 patients with 999 implants were examined clinically and radiographically, to which 48% had probing depths > 4 mm and bleeding on probing, which were diagnosed as peri-implant mucositis [54]. Progressive bone loss was defined as bone loss > 1.8 mm compared to the 1-year recall data, combined with bleeding on probing/suppuration, was diagnosed in 16% of patients and 6.6% of the implants [54]. Other authors observed different figures with reporting a prevalence of 28% at the patient level [55]. Reasons for the discrepancy in prevalence found by this review against other papers can be explained by the lack of consensus in reporting peri-implant diseases. A systematic review and meta-analysis screened the literature for the prevalence of peri-implant mucositis and peri-implantitis, reported that due to the current literature having inconsistencies in definitions for peri-implant diseases, reporting methods and study characteristics, the prevalence of these diseases varies significantly amongst studies [56].

### 4.3. Hardware Complications

Salvi and Bragger were among the first to define mechanical and technical complications in implant therapy, and explored the ways in which certain risk factors can be in association with these complications [57]. This present review explores hardware, both technical and mechanical complications, that have occurred in single IIP. Several reviews have looked at the rates of hardware complications on IIP, however, previous research has tended to refer to hardware complications as technical complications [13,58,59,60]. This is as opposed to distinguishing the latter into a subcategory of the former, making the distinction and comparing these studies in this review difficult. 

The most common technical problems found in this review were the loss of crown retention or loosening and crown fracture. Crown fracture was found in four cases in one study that was assessing the outcomes between immediate loading and delayed loading, with no provisional crown fractures in the delayed group [44]. This is comparable to another systematic review showing crown loosening to be the second most common technical complication, with a reported incidence of 4.3% amongst 2186 single implant supported prostheses. The distinction between mechanical and technical complications were not made in the study conducted by Zembic et al., which reported the most common technical complication being abutment screw loosening, which is defined as a mechanical complication in our study [59]. On the contrary, this review found no incidences of veneer chipping, upon which was demonstrated as the third most common complication in a previous review [59]. Due to the disparity in sample size numbers, any links between studies cannot be inferred from an evidence-based standpoint.

When analyzing mechanical complications, abutment screw loosening was the most common complication in this review—comparable to the Zembic’s study as aforementioned [59]. Another review that looked at IIP found a single incidence of abutment screw loosening as the only hardware complication, making quantifying any conclusions impossible [13]. Other studies that have looked fixed implant rehabilitations for edentulous patients or implant supported bridges also found abutment screw loosening as the most common technical complication, indicating that abutment screw loosening may be a common technical issue in all forms of implant related prostheses, although further rigorous studies may be needed to document this [58,60].

Despite the relatively low incidences of technical complications reported in the present study, there is some evidence that compared with tooth-supported restorations, the incidence of hardware complications in conventionally placed implant supported single crowns has been found to be higher than in tooth-born restorations, with numerous technical complications in implant supported single-crowns [13]. It has been suggested that this may be due to the lack of periodontal ligament around implants due to its ankylotic fusion to the bone, resulting in overloading of the restoration due to a lack of mechanoreceptive responses from the implant [13]. 

### 4.4. Aesthetics Complications

Currently no systematic review reports complication rates for PES/WES/ICAI, however our results are also comparable to other clinical prospective and retrospective studies that report PES/WES in the literature. When looking at unfavorable PES results, reports range from 0–19% [61,62,63,64], for WES: 0–21% [61,62,63,64], and PES/WES combined: 0–24% of cases [64,65,66,67]. Overall, both the results from our study and those in the literature revealed that unfavorable aesthetic outcomes as measured by PES/WES/ICAI, has a large range from 0–24%. The significant range, for which these results are reported, can be explained by the heterogeneity of IIP studies, i.e., the different protocols used, and the different thresholds for what constitutes an unacceptable aesthetic outcome. Two of our assessed studies arbitrarily set this at PES/WES < 6 [37,50], and the remaining one left it unspecified [34]. Unfortunately, only one of the included studies reported ICAI and additionally the study did not separate the score that constituted a complication into PES, WES or ICAI, so nothing definitive can be remarked about the result reported. In terms of whether the protocol being used affected the rate of unfavorable PES/WES/ICAI results or not, two out of three studies that reported complications did not find anything significant. The Slagter et al. study found no statistical difference between IIP plus immediate restoration and IIP plus delay restoration [34], and neither did Zuiderveld et al. between CTG and non-CTG IIP plus immediate restored implants [37]. The study by Migliorati et al., conversely did report a difference when using CTG in IIP plus immediate restored implants [50]. The authors do discuss that despite other studies, the literature failed to find any influence on aesthetic outcome, and one consideration that must be remarked upon in their study was the significant difference the result of CTG had on thin gingival biotypes between their test and control groups [50]. Thin gingival biotypes have been associated with greater changes in midfacial gingival levels than thick gingival biotypes [68], thus it can be considered that CTG could play a more significant role in managing aesthetic outcomes for those with thin gingival biotypes in IIP, although more studies that control for gingival biotypes would need to be conducted for more conclusive results. 

For midfacial gingival levels, most studies reported a mean recession level, which potentially obscured the exact number of unfavorable outcomes. Only one study reported actual complications occurring in 24% of IIP and delayed loaded implants, and 11.5% of IIP and immediately loaded implants. These results are comparable to those in the systematic review by Lang and co-workers, which reported a 20% incidence of aesthetic complications due to buccal soft tissue recession in their analysis of studies that used a IIP and delay loading protocol [13], and also to the previous results that reported 11% of IIP immediately loaded implants that reported with poor gingival aesthetics due to buccal recession after a mean follow up of 4 years [68]. This finding of immediate restoration providing better midfacial gingival aesthetics is supported by the results of De Rouck et al. who found that recession was 2.5–3 times higher in immediate implants with delayed restoration than those with immediate restoration [28]. However, the other comparative study between immediate restoration and delayed restoration by Slagter et al. found no difference in midfacial gingival aesthetics between immediate restoration and delayed restoration [34]. One possible reason for the differing findings may be explained by how Shibli et al. used neighboring teeth to assess midfacial gingival levels [33], whereas Slagter et al. in 2015 used preoperative levels, which may have a recession to begin with, resulting in a lesser increase in recession [34]. Thus, more studies are needed with a standardized methodology for establishing baseline levels before concluding that immediate restoration of IIP provide a better midfacial gingival appearance than delayed restoration in the long term follow up.

In regard to papillary height complications, our review reported only four studies using PIS and of these 14–24% of cases with a papillary fill less than 50% after one year follow up. This contrasts to the results of two other reviews that found all papillae height ended with a PIS score of two or three (i.e., all papillae had more than 50% fill) [3,13]. The difference in the results can be partly explained by the protocol they followed, in which most of the implants placed had guide bone regeneration with autogenous bone grafts, whereas most of the implants placed in the studies for our review did not undergo hard tissue augmentation. Another difference, however, is the differences in the designs of the included studies, where our review included only RCT studies, whereas all papillary fill results in the other reviews were reported from prospective clinical studies. Further RCT studies needed to assess whether there truly is a difference. 

Regardless of the clinical assessment of aesthetic outcome we reported high patient satisfaction across all studies, which is a finding that is common in other reviews and clinical studies in the literature [2,13]. For all but one study, the protocol used for IIP did not make a difference in patient satisfaction. Only the study by Slagter et al. reported a difference in VAS assessment between immediate restoration and delayed restoration, however the authors report that it is not a clinically relevant difference, as a difference greater than 13 points on the VAS is needed for it to be so [34]. It is postulated that the reason why the delayed restoration resulted in higher postoperative satisfaction is that the immediately restored patients experienced immediate satisfaction, whereas those that were in the delayed group had to undergo replacement with a partial denture, which may have affected their final satisfaction scores after having to undergo the initial use of a partial denture. Another study by Kan et al. reported an overall high patient satisfaction with IIP, although overtime issues with buccal recession decreased patient satisfaction [68]. Thus, studies that evaluate patient satisfaction over longer periods of time, allowing for continuing soft tissue changes will be needed.

One major limitation of this systematic review is the limited data and short follow-up period that most of the included studies have. Only one paper exceeded a follow up of 5 years, with the majority of our papers having only a 1-year follow-up. This impacted some of our results as biological complications and issues with patient satisfaction may be underrepresented as they are more likely to only come to light once reviewed after a long period of time. The heterogeneity of the studies also had an impact on the presentation of our results; issues such as different criteria for success and different measurement methods for gingival levels make it difficult for our results to be generalized. Going forward, more RCT studies with longer follow-up periods and more standardized criteria for assessing success, peri-implant disease, and clinical assessment of aesthetic outcomes will be needed to corroborate the results of this systematic review. 

## 5. Conclusions 

In total, nine of the twenty-six papers (35%) included a reported success rate—ranging from 96.7–100%. Relatively few cases of biological complications were reported in this review; only two cases of peri-implant mucositis and no cases of peri-implantitis were reported, likely due to short term follow-up. The most common technical problems were loss of crown retention or loosening and crown fracture. The most common mechanical problem reported in this review was abutment screw loosening. No studies went into detail about how such complications were managed. 

When considering cases of unacceptable PES/WES, poor outcomes occurred in 0–21.3% of cases depending on the protocol. However, different cutoffs were used for what constituted an unacceptable score or not. In the future, a common cutoff threshold for an acceptable score should also be established in the future for PES/WES. Immediate restoration following single IIP appears to have better results for midfacial gingival levels than a delayed restoration method. More RCT studies to show this would be desirable. Patient satisfaction was high for single IIP regardless of clinical evaluation or the implant protocol followed, although ideally studies with a longer follow-up period would be needed. The heterogeneity, relatively short-term follow-up and quality of included publications suggest that caution should be exercised when interpreting the data and that there remains an important need for additional evidence.

## Figures and Tables

**Table 1:** Risk of bias.

Author, Year	Random Sequence Generation	Allocation Concealment	Blinding of Participants and Personnel	Blinding of Outcome Assessment	Incomplete Outcome Data	Selective Reporting	Other Bias
**Block et al. 2009**	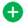	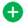	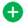	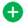	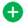	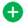	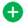
**Cannizzaro et al. 2010**	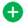	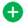	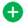	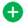	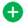	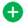	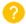
**Canullo et al. 2009/2016**	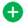	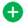	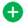	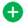	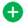	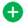	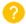
**Canullo et al. 2010**	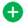	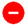	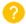	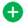	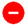	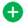	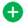
**Cecchinato et al. 2015**	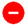	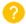	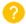	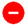	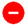	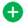	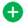
**Cordaro et al. 2009**	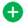	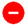	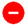	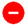	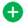	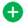	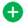
**Crespi et al. 2008**	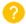	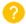	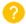	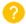	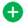	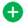	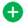
**Cucchi et al. 2017**	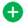	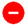	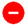	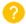	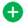	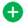	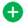
**De Angelis et al. 2011**	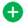	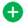	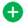	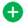	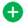	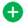	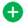
**De Rouck et al. 2009**	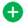	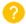	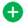	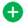	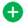	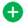	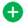
**Degidi et al. 2014**	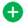	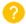	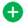	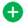	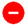	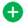	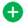
**Esposito et al. 2015**	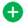	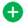	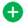	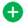	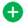	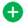	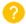
**Felice et al. 2015**	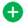	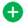	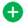	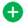	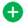	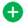	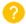
**Grandi et al. 2014**	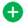	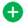	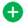	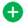	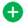	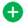	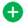
**Koh et al. 2011**	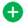	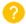	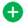	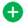	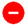	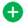	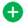
**Migliorati et al. 2013**	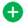	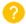	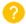	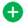	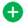	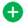	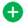
**Nimwegen et al. 2018**	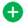	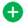	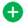	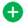	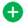	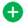	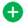
**Palatella et al. 2008**	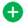	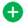	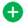	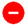	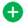	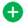	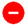
**Pieri et al. 2011**	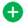	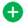	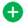	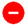	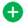	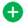	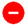
**Prosper et al. 2003**	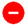	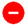	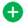	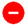	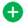	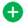	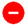
**Shibly, Kutkut and Patel 2010**	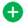	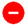	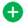	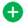	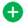	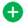	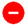
**Slagter et al. 2015**	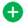	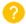	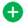	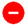	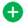	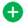	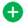
**Tallarico et al. 2016**	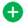	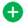	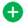	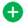	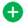	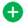	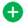
**Urban et al. 2011**	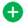	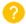	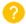	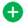	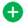	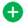	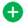
**Yoshino et al. 2014**	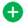	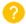	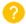	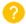	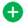	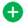	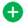
**Zuiderveld et al. 2018**	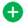	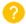	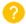	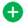	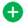	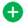	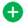

Key: 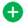 = Low Risk 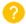 = Unclear Risk 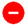 = High Risk.

**Table 2:** Characteristics of Included Studies.

Author and Year	Country	Setting	Population					Intervention	Follow Up (Months)
			Number of Patients and Implants	Male: Female	Mean Age and Range in Years	Region	Reason for Extraction		
Block et al., 2009	USA	-	55/55	-	-	15–25	PD + NPD	Immediate Implant	18–24
Canullo et al., 2010	Italy	Mc, 3 PP	25/25	16:9	Provisional = 51 Definitive = 55	15, 14, 24, 25	NPD	Immediate Implant	36
Canullo et al., 2009/2016	Italy	Mc, 2 PP	22/22 imp platform is 5.5 mm (11 with 3.8 abutment-test and 11 with 5.5 abutment-control) At 10 years, 19/19	13:9	50, 32–76	15–25	-	Immediate Implant	300/120
Cecchinato et al., 2015	Italy	MC	93/93	48:45:00	51 (19–80)	15–25	PD + NPD	Immediate Implant	36
Cordaro et al., 2009	Italy	PP	30/30	-	18–70	15–25, 33–35, 43–45	NPD	Immediate Implant	0, 1.5, 3, 6, 12, 18
Crespi et al., 2008	Italy	U	40/40 = (A = 20 and D = 20)	16:24 A 6:14 D 10:10	47, 24–68		PD + NPD	Immediate Implant	24
Cucchi et al., 2017	Italy	Mc	92/97	43:49:00	51.0 (20–79)	Premolars and Molars	PD + NPD	Immediate Implant	12–36
De Angelis et al., 2011	Italy	Mc (4), PP	80/80	38:42:00	47.05	All regions	-	Immediate Implant	12
De Rouck et al., 2009	Belgium	U	49/49 = (A = 24 and D = 25)	A 11:13 D 12:13	A: 55(13) D: 52(12)	15–25	PD + NPD	Immediate Implant	12
Degidi et al., 2014	Italy	PP	53/53	-	43.9 year	13–23	PD + NPD	Immediate Implant	24
Esposito et al., 2015	Italy	Mc (3) PP	106/106 (54 Type A, D; 52 E, F, placed at 4 months)	22:32 (A, D) 24:28 (E, F)	48 (28–70) for A, D 50 (30–72) for E, F	15–25	-	Immediate Implant	12
Felice et al., 2015	Italy	(4) PP	48/48 (25 A, D; 25 E, F; 2 dropped out at review both from E/F already removed from number^)	12:13 (A, D) 13:12 (E, F)	51.32 (32–71) for A, D 53.08 (39–72) for E, F	15–25	-	Immediate Implant	4, 12
Grandi et al., 2014	Italy	Mc	25/25	9:16	56.54 year (39–74 year)	15–25	PD + NPD?	Immediate Implant	12
Koh et al., 2011	USA	U	20/20	12:8	21–73	15–25	NPD	Immediate Implant	12
Migliorati et al., 2013	Italy	-	48/48	23:25	47.5 (range 22–70)	14–24	NPD	Immediate Implant	0, 0.5 (crown insertions), 12, 24
Palattella et al., 2008	Italy	U	16/18 = (A = 9 and C = 9)	6:10	35	13–23	PD + NPD	Immediate Implant	24
Pieri et al., 2011	Italy	U	38/38 M = 20 and C = 20	15:25 M- 7:13 C- 8:12	45, 26–67	15,14, 24,25	PD + NPD	Immediate Implant	12
Prosper et al., 2003	Italy	U	71/120 (60 A; 60 D) (Single crown restorations)	35:36:00	58.3 (range 26–72)	16–17, 26–27, 36–37, 46–47	PD, NPD	Immediate Implant	3, 6, 9, 12, 24, 26, 48
Shibly et al., 2010	USA	U	60/60 (55 at 1-year)	25:35:00	25–94	18—Max posterior 18—max anterior 18—mnd posterior 1—mnd anterior	PD	Immediate Implant	3, 6, 12
Slagter et al., 2015	Netherlands	U	40/40	13:27	A: 39.4 (19–70) D: 42.3 (22–66)	14–24	Group A: 39.4 year (19–70 year) Group D: 42.3 year (23–66 year)	Immediate Implant	12
Tallarico et al., 2016	Italy	PP	24/24	8:16	53.9 (37–67)	Molar region of maxilla or mandible	53.9 (37–67)	Immediate Implant	12
Tallarico et al., 2017	Italy	PP	24/24	8:16	37–67	Maxillary and mandibular molars	37–67	Immediate Implant	12
Urban et al., 2011	Denmark	-	92/92 (76 at follow up)	48:44:00	50 (23–77)	Molar region of Mnd and Mx	50 years (range 23–77 years)	Immediate Implant	12
Van Nimwegen et al., 2018	Netherlands	U	60/60	28:32:00	46.6 (19.5–82.2)	14–24	46.6 year (19.5–82.2 year)	Immediate Implant	12
Yoshino et al., 2014	USA	U	20/20 (10 vs. 10)	7:13	52.6 (27–87)	15–25	52.6, 27–87	Immediate Implant	12
Zuiderveld et al., 2018	Netherlands	U	60/60	28:32:00	46.7 (19.5–82.2)	14–24	46.7 year (19.5–82.2)	Immediate Implant	12

IP: immediate placement, IDP: immediate-delay placement, DP: delayed placement, IR: immediate restoration, IL: immediate loading, A = IP + IR, A* = IP + IR (2 different abutment designs), C = type II placement + IR, D = IP + DL, E = DP with socket preservation+ IR, F = DP+ DL, GBR: guided bone regeneration, SCTG: subepithelium connective tissue graft, Mc: multicentre, U: university, PP: private practice, SPP: specialist private practice.

**Table 3:** Outcomes of the included studies.

Reference	Comparison	Outcomes				
		Success Criteria	Success Rate	Aesthetic Complications	Technical Complications	Biological Complications
Block et al., 2009	A vs. E, A = 26 and E = 29			-	-	
Canullo et al., 2010	A* (provisional abutment vs. definitive abutment), Provisional = 10, Definitive = 15	Self-Defined success criteria: if implant remained in function and not need to be substituted	100%	-	-	-
Canullo et al., 2009/2016	A* (Platform switching vs. standard restoration), Platform switching = 11, Standard = 11			-	-	-
Cecchinato et al., 2015	D vs. D (cylindrical vs. conical/cylindrical implant), Cylindrical = 45, Conical/cylindrical = 48			40/164 (24.4%) of all sites has a PIS of 0 or 1 17 mesial papilla and 23 distal papilla	-	-
Cordaro et al., 2009	D (submerged vs. non-submerged), submerged = 14, non-submerged = 16			-	-	-
Crespi et al., 2008	A (IL) vs. D (IL), A = 20, D = 20			-	4 occlusal screws became unscrewed in the provisional plastic abutments	-
Cucchi et al., 2017	D, F D = 48, F = 44	Albrektsson et al. (1986) criteria	100%	-	-	-
De Angelis et al., 2011	D, GBR *n* = 80 GBR = 40, GBR + BS = 40			-	For the GBR group: 1 incidence of loosening of the cover screw at 4-6 weeks postoperatively, as well as decementation of the final crown of an implant in position 25. For the GBR + BS (Bone substitute): 1 incidence Loosening of the provisional abutment (position 26), 1 incidence of loosening of the cover screw at 4/6 weeks post-operatively (position 36)	1 case of a small lesion in the peri-implant mucosa of tooth 25. 1 case of peri-implant mucositis on tooth 36.
De Rouck et al., 2009	A vs. D A = 24, D = 25			-	-	-
Degidi et al., 2014	A (non removal of abutments vs. standard removal protocol) Test = 24 Control = 29			-	-	1 case of edema at surgery site with loss of thermal sensitivity in the 3-3 region.
Esposito et al., 2015	A, D vs. E, F (both socket preservation), GBR			-	The complications in A,D were partial fracture of the provisional crown (four patients); loosening of the provisional crowns (two patients)	-
Felice et al., 2015	A, D vs. E, F, GBR A + D = 54 E + F = 52			-	Loosening of the provisional 1 crown 3 months after loading for A,D. Loosening of provisional crown in 2 patients, 1 and 3 months after loading for E + F.	-
Grandi et al., 2014	A* (definitive abutment vs. provisional abutment) DA = 12, PA = 13			-	One patient in the PA group had an abutment screw loosening 3 weeks after healing. 4 patients in the DA group had excess cement that had to be removed prior to final crown cementation	1 case of peri-implant mucositis in the 5-5 region.
Koh et al., 2011	D *n* = 24	Misch et al. (2008)—ICOI Pisa Consensus Conference Criteria Self-Defined success criteria: individual implants exhibiting 1.5 mm bone remodelling and thereafter 0.2 mm annually. Lack of mobility, persistent infection, pain or was removed.	-	-	-	-
Migliorati et al., 2013	A, SCTG vs. non-SCTG SCTG = 24 non SCTG = 24			17.3% (*n* = 4) of sites resulted in a poor aesthetic outcome (PES < 6) in a control group consisting of ungrafted immediate implants, Whereas the grafted test group reported no unacceptable aesthetic outcomes.	-	-
Palattella et al., 2008	A vs. C A = 9, C = 8			5/36 (13.9%) total sites has a PIS of 0 or 1 3/18 (16.7%) of patients in the IP + IR group 2/18 (11.7%) in the IP + DR group. Note results were not split into mesial and distal papilla. DIfference not statistically significant.	-	-
Pieri et al., 2011	A*, (Morse-Taper vs. conventional) Morse-taper = 20 Conventional = 20	Smith and Zarb (1989) criteria	97.4% for test group, 100% in the control group	-	Control group: one abutment screw loosening 2 months after delivery of definitive crown (6 months after implant placement). One other patient had a provisional crown fracture after 3 weeks. This was replaced within 24 h	-
Prosper et al., 2003	D, (Hydroxyapatite vs. Resorbable Membrane). Synthetic hydroxyapatite (HA) = 56 vs. membrane (MR) = 55	Albrektsson (1986) criteria	98.2% for implants placed with resorbable synthetic hydroxyapatite (HA), 96.4% for implants placed with a resorbable membrane (MR). This leads to an overall success rate of 97.3%	-	-	-
Shibly et al., 2010	A(IL) v D A = 30, D = 30			7/29 (24.1%)implants had a decreased attached gingiva in group D, 4 of which needed corrective mucogingival surgery versus 3/26 (11.5%) implants in group A(IL) with decreased attached gingiva, of which 1 needed mucogingival surgery.	-	1 implant in the conventional loading group failed due to an acute infection within 2 weeks of replacement implant (mandibular second premolar).
Slagter et al., 2015	A vs. D A = 20, D = 20	Self-Defined success criteria: Clinically stable and fulfilled their function without any discomfort to the patient for 1 year	-	Clinically unsatisfactory ICAI and PES/WES scores were found in a total of 6% of patients (*n* = 2) across both groups, with no significant difference between the scores of the groups	-	-
Tallarico et al., 2016	D v F (with socket preservation) D = 12, F = 12			-	-	-
Tallarico et al., 2017	D,F with socket preservation in both D = 12, F = 12			-	-	-
Urban et al., 2011	D (autologous bone chips, ossix membrane, combination) AB = 26, OM = 28, ABOM = 23			-	-	-
Van Nimwegen et al., 2018	A (SCTG vs. No SCTG) SCTG = 30, No SCTG = 30			-	-	-
Yoshino et al., 2014	A (SCTG vs. No SCTG) SCTG = 10, No SCTG = 10	Smith and Zarb (1989) criteria	100%	9/20 (22.5%) of total sites had a PIS of 0 or 1. Control group = 1 mesial papilla and 3 distal papilla Test group = 3 mesial papilla and 2 distal papilla No statistical difference between groups	1 episode of provisional restoration debonding. 1 provisional restoration fractured near the cervical aspect during removal at the time of final impression making	-
Zuiderveld et al., 2018	A (SCTG vs. no SCTG) SCTG = 30, No SCTG = 30 Note one implant in each group lost due to failure of survival	Albrektsson et al. (1986) criteria	96.7%	Zuiderveld et al. (2018) reported 21.3% (12/58)of cases with an unacceptable level of aesthetics (PES <6) for the peri-implant mucosa and 14.9% (8/58) for the implant crown aesthetics (WES < 6) across both groups, with no difference in PES/WES scores between groups. 15.5% (9/58) of total sites had a PIS of 0 or 1.No difference in scores in between Control and test Control group: 3 mesial papilla and 2 distal papilla Test group: 2 mesial papilla and 2 distal papilla	-	-

IP: immediate placement, IDP: immediate-delay placement, DP: delayed placement, IR: immediate restoration, IL: immediate loading, A = IP + IR, A* = IP + IR (2 different abutment designs), C = type II placement + IR, D = IP + DL, E = DP with socket preservation + IR, F = DP+ DL, GBR: guided bone regeneration, SCTG: subepithelium connective tissue graft, Mc: multicentre, U: university, PP: private practice, SPP: specialist private practice.
